# [2-({3-[(3-Amino­prop­yl)amino]­prop­yl}imino­meth­yl)phenolato-κ^4^ *O*,*N*,*N*′′,*N*′′′]bromidocopper(II)

**DOI:** 10.1107/S1600536810042923

**Published:** 2010-10-30

**Authors:** Gervas E. Assey, Anand M. Butcher, Ray J. Butcher, Yilma Gultneh

**Affiliations:** a Department of Chemistry, Howard University, 525 College Street NW, Washington, DC 20059, USA

## Abstract

In the title compound, [Cu(C_13_H_20_N_3_O)Br], the Cu(II) atom is coordinated by three N atoms and one O atom from the deprotonated ligand derived from the Schiff base condensation of 3,3-imino­bis­(propyl­amine) and salicyl­aldehyde. The three N and the O atoms occupy equatorial positions, while the Br atom occupies an axial position. The amine H atoms form inter­molecular hydrogen bonds with the Br and O atoms of adjoining mol­ecules

## Related literature

For asymmetry parameters, see: Addison *et al.* (1984 ▶). For the preparation of the ligand, see: Pajunen *et al.* (2000 ▶).
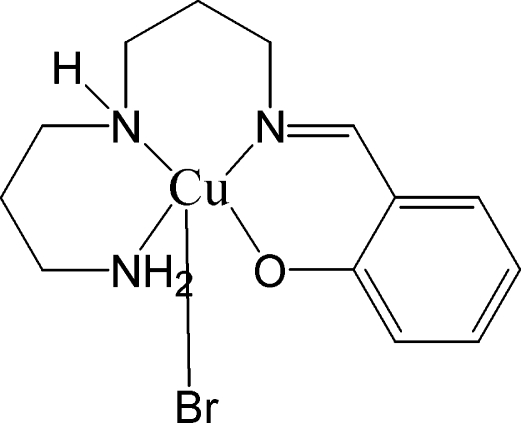


## Experimental

### 

#### Crystal data

[Cu(C_13_H_20_N_3_O)Br]
*M* *_r_* = 377.77
Orthorhombic, 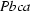

*a* = 12.3272 (2) Å
*b* = 11.34425 (19) Å
*c* = 20.5729 (4) Å
*V* = 2876.98 (9) Å^3^
*Z* = 8
Cu *K*α radiation
μ = 5.36 mm^−1^
*T* = 173 K
0.44 × 0.23 × 0.07 mm

#### Data collection

Oxford Diffraction Xcalibur Ruby Gemini diffractometer
Absorption correction: analytical [*CrysAlis RED* (Oxford Diffraction, 2007 ▶); based on Clark & Reid (1995 ▶)] *T*_min_ = 0.211, *T*_max_ = 0.697
8196 measured reflections
3021 independent reflections
2939 reflections with *I* > 2σ(*I*)
*R*_int_ = 0.024

#### Refinement

*R*[*F* ^2^ > 2σ(*F* ^2^)] = 0.039
*wR*(*F* ^2^) = 0.096
*S* = 1.09
3021 reflections
172 parameters
H-atom parameters constrained
Δρ_max_ = 1.19 e Å^−3^
Δρ_min_ = −0.77 e Å^−3^

### 

Data collection: *CrysAlis PRO* (Oxford Diffraction, 2007 ▶); cell refinement: *CrysAlis PRO*; data reduction: *CrysAlis PRO*; program(s) used to solve structure: *SHELXS97* (Sheldrick, 2008 ▶); program(s) used to refine structure: *SHELXL97* (Sheldrick, 2008 ▶); molecular graphics: *SHELXTL* (Sheldrick, 2008 ▶); software used to prepare material for publication: *SHELXTL*.

## Supplementary Material

Crystal structure: contains datablocks I, global. DOI: 10.1107/S1600536810042923/pb2044sup1.cif

Structure factors: contains datablocks I. DOI: 10.1107/S1600536810042923/pb2044Isup2.hkl

Additional supplementary materials:  crystallographic information; 3D view; checkCIF report

## Figures and Tables

**Table:** 

Cu—O1	1.943 (2)
Cu—N1	1.998 (3)
Cu—N3	2.029 (3)
Cu—N2	2.061 (3)
Cu—Br	2.8555 (5)

**Table:** 

O1—Cu—N1	90.84 (10)
O1—Cu—N3	82.43 (10)
N1—Cu—N3	167.76 (11)
O1—Cu—N2	165.04 (11)
N1—Cu—N2	95.97 (11)
N3—Cu—N2	88.36 (11)
O1—Cu—Br	99.26 (7)
N1—Cu—Br	98.38 (8)
N3—Cu—Br	92.81 (8)
N2—Cu—Br	92.92 (8)

**Table 2:** Hydrogen-bond geometry (Å, °)

*D*—H⋯*A*	*D*—H	H⋯*A*	*D*⋯*A*	*D*—H⋯*A*
N2—H2*B*⋯Br^i^	0.91	2.62	3.472 (3)	157
N3—H3*B*⋯O1^ii^	0.90	2.16	2.938 (3)	144
N3—H3*C*⋯Br^ii^	0.90	2.65	3.488 (3)	156

Symmetry codes: (i) 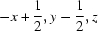; (ii) 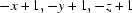.

**Fig. 1.**
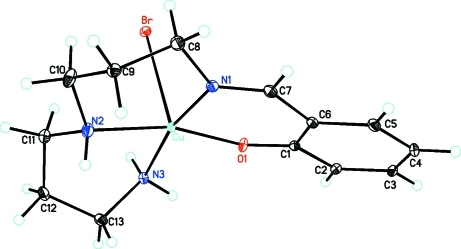
A view of the title compound, C13H20BrCuN3O, showing atom numbering scheme.

**Fig. 2.**
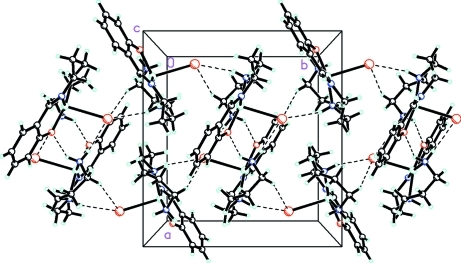
The molecular packing for C13H20BrCuN3O, viewed down the c axis showing the intermolecular N—H···O and N—H···Br interactions as dashed lines.

## supplementary crystallographic information

### Comment

The stucture of the title compound, (I), is shown below. Dimensions are available in the archived CIF.

The reported structure is related to a previously published structure that contains a mononuclear copper(II) complex of a Schiff base resulting from the condensation of an imidazole-aldehyde with 3,3-iminobispropylamine (Pajunen *et al.*, 2000). In this paper we report the synthesis of a new copper(II) complex containing a phenolato ligand in place of the imidazole. As in the latter case, while the reaction was carried out with an amine:salicylaldehyde ratio of 1:2, the resulting Schiff base ligand was the condensation product of one salicylaldehyde molecule and one amine molecule thus the ligand contains one imino and two amine N's. One difference between the copper complexes of the two ligands is that the copper(II) complex of the imidazole ligand is a cation with methanol as one of the ligands and an uncoordinated perchlorate anion while the title compound contains coordinated Br^-^ and is thus neutral.

In the title compound C_13_H_20_BrCuN_3_O, the Cu is penta-coordinated with the phenolic O and N atoms forming a plane and with an axial bromide anion and the Cu 0.205 (1) Å out of the basal plane. Thus the overall geometry is square pyramidal [τ = 0.045 (Addison *et al.*, 1984)]. The bond distance between Cu(II) and the phenolic O is 1.943 (2) Å which is shorter than the Cu—N distances involving the amine N's, *i.e.*, Cu N1 1.998 (3); Cu N3 2.029 (3); Cu N2 2.061 (3) Å. The apical Cu—Br distance is 2.8555 (5) Å.

The amine protons form intermolecular hydrogen bonds with the Br and O atoms of adjoining molecules.

### Experimental

The synthesis of the 3,3'-iminobis(propylamine)salicylaldimine was accomplished by the reaction of a solution of (5 g, 37.34 mmol) 3,3-iminobispropylamine in 20 ml methanol with a solution of (9.13 g, 74.68 mmol) salicylaldehyde in 40 ml methanol. The reaction mixture was refluxed for 24 h and then evaporated under reduced pressure to give a brownish yellow oily liquid.

The complex was synthesized by mixing a solution of 3,3'-iminobis(propylamine)salicylaldehyde (0.25 g, 0.74 mmol) in 10 ml methanol to a solution of (0.21 g, 1.48 mmol) CuBr in 10 ml methanol. The mixture was stirred for 24 h at room temperature. At the end of the reaction, the reaction mixture was evaporated under reduced pressure to afford greenish solids. The solids were dissolved in DMF and filtered. The filtrate solution of the complex was layered with diethyl ether for slow solvent diffusion crystallization method. Crystals suitable for X-ray diffraction were obtained.

### Refinement

H atoms were placed in geometrically idealized positions and constrained to ride on their parent atoms with a C—H distances of 0.93 and 0.97 Å [*U*_iso_(H) = 1.2*U*_eq_(C)]. The H atoms attached to N were idealized with an primary and secondary N–H distances of 0.90 and 0.91 Å, respectively.

### Crystal data

**Table:**

[Cu(C_13_H_20_N_3_O)Br]	*F*(000) = 1528
*M**_r_* = 377.77	*D*_x_ = 1.744 Mg m^−^^3^
Orthorhombic, *P**b**c**a*	Cu *K*α radiation, λ = 1.54184 Å
Hall symbol: -P 2ac 2ab	Cell parameters from 6661 reflections
*a* = 12.3272 (2) Å	θ = 4.2–77.1°
*b* = 11.34425 (19) Å	µ = 5.36 mm^−^^1^
*c* = 20.5729 (4) Å	*T* = 173 K
*V* = 2876.98 (9) Å^3^	Plate, blue
*Z* = 8	0.44 × 0.23 × 0.07 mm

### Data collection

**Table:**

Oxford Diffraction Xcalibur Ruby Gemini diffractometer	3021 independent reflections
Radiation source: fine-focus sealed tube	2939 reflections with *I* > 2σ(*I*)
graphite	*R*_int_ = 0.024
Detector resolution: 10.5081 pixels mm^-1^	θ_max_ = 77.5°, θ_min_ = 5.7°
ω scans	*h* = −14→15
Absorption correction: analytical [*CrysAlis RED* (Oxford Diffraction, 2007); based on expressions derived by Clark & Reid (1995)]	*k* = −14→8
*T*_min_ = 0.211, *T*_max_ = 0.697	*l* = −25→15
8196 measured reflections	

### Refinement

**Table:**

Refinement on *F*^2^	Primary atom site location: structure-invariant direct methods
Least-squares matrix: full	Secondary atom site location: difference Fourier map
*R*[*F*^2^ > 2σ(*F*^2^)] = 0.039	Hydrogen site location: inferred from neighbouring sites
*wR*(*F*^2^) = 0.096	H-atom parameters constrained
*S* = 1.09	*w* = 1/[σ^2^(*F*_o_^2^) + (0.0427*P*)^2^ + 9.8873*P*] where *P* = (*F*_o_^2^ + 2*F*_c_^2^)/3
3021 reflections	(Δ/σ)_max_ = 0.002
172 parameters	Δρ_max_ = 1.19 e Å^−^^3^
0 restraints	Δρ_min_ = −0.77 e Å^−^^3^

### Special details

**Table:**

Experimental. Absorption correction: CrysAlis RED (Oxford Diffraction, 2007). Analytical numeric absorption correction using a multifaceted crystal model based on expressions derived by Clark & Reid. [Clark, R. C. & Reid, J. S. (1995). Acta Cryst. A51, 887-897]
Geometry. All e.s.d.'s (except the e.s.d. in the dihedral angle between two l.s. planes) are estimated using the full covariance matrix. The cell e.s.d.'s are taken into account individually in the estimation of e.s.d.'s in distances, angles and torsion angles; correlations between e.s.d.'s in cell parameters are only used when they are defined by crystal symmetry. An approximate (isotropic) treatment of cell e.s.d.'s is used for estimating e.s.d.'s involving l.s. planes.
Refinement. Refinement of *F*^2^ against ALL reflections. The weighted *R*-factor *wR* and goodness of fit *S* are based on *F*^2^, conventional *R*-factors *R* are based on *F*, with *F* set to zero for negative *F*^2^. The threshold expression of *F*^2^ > σ(*F*^2^) is used only for calculating *R*-factors(gt) *etc*. and is not relevant to the choice of reflections for refinement. *R*-factors based on *F*^2^ are statistically about twice as large as those based on *F*, and *R*- factors based on ALL data will be even larger.

### Fractional atomic coordinates and isotropic or equivalent isotropic displacement parameters (Å^2^)

**Table:**

	*x*	*y*	*z*	*U*_iso_*/*U*_eq_	
Cu	0.32735 (4)	0.48683 (4)	0.57075 (2)	0.01378 (13)	
Br	0.39440 (3)	0.72635 (3)	0.556355 (16)	0.02033 (12)	
O1	0.46552 (18)	0.4169 (2)	0.59480 (10)	0.0186 (4)	
N1	0.2822 (2)	0.4932 (2)	0.66398 (12)	0.0161 (5)	
N2	0.1756 (2)	0.5198 (3)	0.53280 (14)	0.0229 (6)	
H2B	0.1410	0.4490	0.5305	0.027*	
N3	0.3774 (2)	0.4431 (2)	0.47993 (12)	0.0171 (5)	
H3B	0.4019	0.5089	0.4604	0.021*	
H3C	0.4339	0.3934	0.4836	0.021*	
C1	0.4937 (2)	0.3662 (3)	0.64894 (14)	0.0149 (6)	
C2	0.5933 (3)	0.3039 (3)	0.65136 (15)	0.0173 (6)	
H2A	0.6367	0.3000	0.6144	0.021*	
C3	0.6269 (3)	0.2487 (3)	0.70795 (17)	0.0202 (6)	
H3A	0.6927	0.2087	0.7084	0.024*	
C4	0.5638 (3)	0.2521 (3)	0.76433 (15)	0.0224 (7)	
H4A	0.5866	0.2142	0.8020	0.027*	
C5	0.4670 (3)	0.3130 (3)	0.76273 (15)	0.0204 (6)	
H5A	0.4245	0.3159	0.8001	0.025*	
C6	0.4306 (3)	0.3708 (3)	0.70627 (15)	0.0166 (6)	
C7	0.3314 (3)	0.4381 (3)	0.71007 (15)	0.0181 (6)	
H7A	0.2990	0.4421	0.7508	0.022*	
C8	0.1888 (3)	0.5655 (3)	0.68384 (17)	0.0251 (7)	
H8A	0.2099	0.6479	0.6833	0.030*	
H8B	0.1691	0.5453	0.7281	0.030*	
C9	0.0905 (3)	0.5495 (3)	0.64062 (16)	0.0197 (6)	
H9A	0.0738	0.4661	0.6376	0.024*	
H9B	0.0288	0.5884	0.6606	0.024*	
C10	0.1058 (3)	0.5980 (3)	0.57295 (18)	0.0276 (8)	
H10A	0.0355	0.6064	0.5522	0.033*	
H10B	0.1386	0.6755	0.5756	0.033*	
C11	0.1810 (3)	0.5657 (3)	0.46581 (17)	0.0253 (7)	
H11A	0.2339	0.6290	0.4644	0.030*	
H11B	0.1110	0.5991	0.4546	0.030*	
C12	0.2108 (3)	0.4752 (3)	0.41555 (16)	0.0227 (7)	
H12A	0.2365	0.5156	0.3770	0.027*	
H12B	0.1460	0.4318	0.4036	0.027*	
C13	0.2965 (3)	0.3883 (3)	0.43705 (15)	0.0213 (6)	
H13A	0.2621	0.3235	0.4598	0.026*	
H13B	0.3328	0.3565	0.3991	0.026*	

### Atomic displacement parameters (Å^2^)

**Table:**

	*U*^11^	*U*^22^	*U*^33^	*U*^12^	*U*^13^	*U*^23^
Cu	0.0140 (2)	0.0144 (2)	0.0130 (2)	0.00279 (16)	−0.00072 (16)	0.00046 (15)
Br	0.0271 (2)	0.01531 (18)	0.01858 (18)	0.00442 (12)	−0.00268 (12)	−0.00103 (11)
O1	0.0161 (10)	0.0234 (11)	0.0162 (10)	0.0068 (9)	0.0011 (8)	0.0063 (8)
N1	0.0150 (12)	0.0182 (12)	0.0151 (12)	0.0019 (10)	0.0023 (10)	−0.0021 (9)
N2	0.0202 (14)	0.0256 (14)	0.0228 (14)	0.0046 (11)	−0.0023 (11)	0.0012 (11)
N3	0.0168 (12)	0.0193 (13)	0.0151 (12)	0.0006 (10)	−0.0021 (10)	0.0015 (10)
C1	0.0166 (14)	0.0123 (13)	0.0159 (13)	−0.0015 (11)	−0.0025 (11)	0.0003 (10)
C2	0.0187 (15)	0.0150 (14)	0.0183 (14)	0.0009 (12)	−0.0012 (11)	0.0004 (12)
C3	0.0204 (15)	0.0158 (13)	0.0243 (16)	0.0038 (12)	−0.0088 (13)	−0.0003 (12)
C4	0.0300 (18)	0.0215 (14)	0.0155 (15)	0.0035 (14)	−0.0086 (13)	0.0015 (12)
C5	0.0265 (16)	0.0212 (15)	0.0135 (14)	−0.0010 (13)	−0.0008 (12)	−0.0014 (11)
C6	0.0166 (14)	0.0163 (14)	0.0168 (13)	−0.0021 (11)	−0.0032 (11)	−0.0020 (11)
C7	0.0194 (15)	0.0215 (15)	0.0135 (13)	−0.0010 (12)	0.0027 (11)	−0.0025 (11)
C8	0.0217 (16)	0.0308 (17)	0.0228 (16)	0.0092 (14)	−0.0010 (13)	−0.0071 (14)
C9	0.0148 (14)	0.0206 (15)	0.0238 (15)	0.0016 (12)	0.0020 (12)	−0.0021 (12)
C10	0.0206 (17)	0.0331 (19)	0.0292 (18)	0.0112 (14)	0.0036 (13)	0.0079 (15)
C11	0.0262 (17)	0.0233 (16)	0.0262 (17)	0.0038 (14)	−0.0072 (14)	0.0013 (14)
C12	0.0211 (16)	0.0259 (17)	0.0211 (15)	−0.0044 (13)	−0.0072 (13)	0.0032 (13)
C13	0.0214 (15)	0.0247 (16)	0.0180 (14)	−0.0010 (13)	0.0007 (12)	−0.0054 (12)

### Geometric parameters (Å, °)

**Table:**

Cu—O1	1.943 (2)	C4—H4A	0.9300
Cu—N1	1.998 (3)	C5—C6	1.407 (4)
Cu—N3	2.029 (3)	C5—H5A	0.9300
Cu—N2	2.061 (3)	C6—C7	1.444 (4)
Cu—Br	2.8555 (5)	C7—H7A	0.9300
O1—C1	1.301 (4)	C8—C9	1.514 (5)
N1—C7	1.288 (4)	C8—H8A	0.9700
N1—C8	1.472 (4)	C8—H8B	0.9700
N2—C11	1.475 (4)	C9—C10	1.508 (5)
N2—C10	1.487 (4)	C9—H9A	0.9700
N2—H2B	0.9100	C9—H9B	0.9700
N3—C13	1.469 (4)	C10—H10A	0.9700
N3—H3B	0.9000	C10—H10B	0.9700
N3—H3C	0.9000	C11—C12	1.503 (5)
C1—C6	1.414 (4)	C11—H11A	0.9700
C1—C2	1.418 (4)	C11—H11B	0.9700
C2—C3	1.385 (4)	C12—C13	1.511 (5)
C2—H2A	0.9300	C12—H12A	0.9700
C3—C4	1.397 (5)	C12—H12B	0.9700
C3—H3A	0.9300	C13—H13A	0.9700
C4—C5	1.379 (5)	C13—H13B	0.9700
			
O1—Cu—N1	90.84 (10)	C5—C6—C7	118.1 (3)
O1—Cu—N3	82.43 (10)	C1—C6—C7	122.0 (3)
N1—Cu—N3	167.76 (11)	N1—C7—C6	128.0 (3)
O1—Cu—N2	165.04 (11)	N1—C7—H7A	116.0
N1—Cu—N2	95.97 (11)	C6—C7—H7A	116.0
N3—Cu—N2	88.36 (11)	N1—C8—C9	113.4 (3)
O1—Cu—Br	99.26 (7)	N1—C8—H8A	108.9
N1—Cu—Br	98.38 (8)	C9—C8—H8A	108.9
N3—Cu—Br	92.81 (8)	N1—C8—H8B	108.9
N2—Cu—Br	92.92 (8)	C9—C8—H8B	108.9
C1—O1—Cu	129.3 (2)	H8A—C8—H8B	107.7
C7—N1—C8	115.8 (3)	C10—C9—C8	113.5 (3)
C7—N1—Cu	123.9 (2)	C10—C9—H9A	108.9
C8—N1—Cu	120.3 (2)	C8—C9—H9A	108.9
C11—N2—C10	109.5 (3)	C10—C9—H9B	108.9
C11—N2—Cu	112.1 (2)	C8—C9—H9B	108.9
C10—N2—Cu	115.0 (2)	H9A—C9—H9B	107.7
C11—N2—H2B	106.5	N2—C10—C9	111.6 (3)
C10—N2—H2B	106.5	N2—C10—H10A	109.3
Cu—N2—H2B	106.5	C9—C10—H10A	109.3
C13—N3—Cu	116.8 (2)	N2—C10—H10B	109.3
C13—N3—H3B	108.1	C9—C10—H10B	109.3
Cu—N3—H3B	108.1	H10A—C10—H10B	108.0
C13—N3—H3C	108.1	N2—C11—C12	114.3 (3)
Cu—N3—H3C	108.1	N2—C11—H11A	108.7
H3B—N3—H3C	107.3	C12—C11—H11A	108.7
O1—C1—C6	123.4 (3)	N2—C11—H11B	108.7
O1—C1—C2	118.8 (3)	C12—C11—H11B	108.7
C6—C1—C2	117.7 (3)	H11A—C11—H11B	107.6
C3—C2—C1	120.9 (3)	C11—C12—C13	114.5 (3)
C3—C2—H2A	119.6	C11—C12—H12A	108.6
C1—C2—H2A	119.6	C13—C12—H12A	108.6
C2—C3—C4	121.3 (3)	C11—C12—H12B	108.6
C2—C3—H3A	119.3	C13—C12—H12B	108.6
C4—C3—H3A	119.3	H12A—C12—H12B	107.6
C5—C4—C3	118.4 (3)	N3—C13—C12	112.0 (3)
C5—C4—H4A	120.8	N3—C13—H13A	109.2
C3—C4—H4A	120.8	C12—C13—H13A	109.2
C4—C5—C6	121.9 (3)	N3—C13—H13B	109.2
C4—C5—H5A	119.0	C12—C13—H13B	109.2
C6—C5—H5A	119.0	H13A—C13—H13B	107.9
C5—C6—C1	119.8 (3)		
			
N1—Cu—O1—C1	17.0 (3)	O1—C1—C2—C3	179.8 (3)
N3—Cu—O1—C1	−152.8 (3)	C6—C1—C2—C3	0.5 (4)
N2—Cu—O1—C1	−100.3 (5)	C1—C2—C3—C4	0.3 (5)
Br—Cu—O1—C1	115.6 (2)	C2—C3—C4—C5	−0.6 (5)
O1—Cu—N1—C7	−13.5 (3)	C3—C4—C5—C6	0.2 (5)
N3—Cu—N1—C7	42.8 (6)	C4—C5—C6—C1	0.6 (5)
N2—Cu—N1—C7	153.1 (3)	C4—C5—C6—C7	−176.5 (3)
Br—Cu—N1—C7	−113.0 (3)	O1—C1—C6—C5	179.8 (3)
O1—Cu—N1—C8	165.4 (2)	C2—C1—C6—C5	−0.9 (4)
N3—Cu—N1—C8	−138.2 (5)	O1—C1—C6—C7	−3.3 (5)
N2—Cu—N1—C8	−27.9 (3)	C2—C1—C6—C7	176.0 (3)
Br—Cu—N1—C8	65.9 (2)	C8—N1—C7—C6	−173.4 (3)
O1—Cu—N2—C11	−84.8 (5)	Cu—N1—C7—C6	5.6 (5)
N1—Cu—N2—C11	158.5 (2)	C5—C6—C7—N1	−177.0 (3)
N3—Cu—N2—C11	−33.0 (2)	C1—C6—C7—N1	6.0 (5)
Br—Cu—N2—C11	59.8 (2)	C7—N1—C8—C9	−134.5 (3)
O1—Cu—N2—C10	149.1 (4)	Cu—N1—C8—C9	46.5 (4)
N1—Cu—N2—C10	32.5 (3)	N1—C8—C9—C10	−68.5 (4)
N3—Cu—N2—C10	−159.0 (2)	C11—N2—C10—C9	175.5 (3)
Br—Cu—N2—C10	−66.3 (2)	Cu—N2—C10—C9	−57.1 (4)
O1—Cu—N3—C13	135.8 (2)	C8—C9—C10—N2	75.6 (4)
N1—Cu—N3—C13	78.7 (6)	C10—N2—C11—C12	−157.6 (3)
N2—Cu—N3—C13	−32.4 (2)	Cu—N2—C11—C12	73.4 (3)
Br—Cu—N3—C13	−125.2 (2)	N2—C11—C12—C13	−39.1 (4)
Cu—O1—C1—C6	−11.3 (4)	Cu—N3—C13—C12	70.9 (3)
Cu—O1—C1—C2	169.3 (2)	C11—C12—C13—N3	−34.9 (4)

### Hydrogen-bond geometry (Å, °)

**Table:**

*D*—H···*A*	*D*—H	H···*A*	*D*···*A*	*D*—H···*A*
N2—H2B···Br^i^	0.91	2.62	3.472 (3)	157
N3—H3B···O1^ii^	0.90	2.16	2.938 (3)	144
N3—H3C···Br^ii^	0.90	2.65	3.488 (3)	156

Symmetry codes: (i) −*x*+1/2, *y*−1/2, *z*; (ii) −*x*+1, −*y*+1, −*z*+1.
